# Antianhedonic Effect of Repeated Ketamine Infusions in Patients With Treatment Resistant Depression

**DOI:** 10.3389/fpsyt.2021.704330

**Published:** 2021-10-18

**Authors:** Alina Wilkowska, Mariusz Stanisław Wiglusz, Maria Gałuszko-Wegielnik, Adam Włodarczyk, Wiesław Jerzy Cubała

**Affiliations:** Department of Psychiatry, Faculty of Medicine, Medical University of Gdańsk, Gdańsk, Poland

**Keywords:** ketamine, anhedonia, treatment resistant depression, benzodiazepines, antianhedonic

## Abstract

Anhedonia constitutes one of the main symptoms of depressive episode. It correlates with suicidality and significantly effects the quality of patient's lives. Available treatments are not sufficient against this group of symptoms. Ketamine is a novel, rapid acting strategy for treatment resistant depression. Here we report the change in symptoms of anhedonia measured by Snaith-Hamilton Pleasure Scale as an effect of eight ketamine infusions as an add-on treatment in 42 patients with treatment resistant depression. We also determined the effect of this change on the severity of depressive symptoms measured by Inventory for Depression Symptomatology-Self Report 30-Item (IDS-SR 30). We have observed statistically significant decrease in the level of anhedonia during ketamine treatment. After adjusting for potential confounders we have found that significant reduction in Snaith-Hamilton Pleasure Scale (SHAPS) after each infusion and 1 week post treatment was observed only among patients who did not use benzodiazepines. The reduction in symptoms of anhedonia mediates the antidepressive effect of ketamine. The results need replication in a larger randomized placebo controlled trial.

## Introduction

Anhedonia, the reduction of the ability to experience pleasure, is one of the core symptoms of depression, and approved treatments do not address it sufficiently (1). The presence of anhedonia strongly correlates with suicidality, and this effect is independent of the severity of depressive symptoms (2). It has been shown that anhedonia is a risk factor of completed suicide during the 1-year follow-up (3). Symptoms of anhedonia turned out to be a robust predictor of a poor outcome of antidepressant treatment in a factor analysis of data from two large studies: Genome-based Therapeutic Drugs for Depression (GENDEP) and Sequenced Treatment Alternatives to Relieve Depression (STAR*D). This was true irrespective of which antidepressant was used and did not depend on the level of baseline depression (4). Ketamine is a novel, effective, and rapid-acting antidepressant in unipolar patients (5, 6) and bipolar treatment resistant depression (7). It has also antisuicidal properties (8). Ketamine is an N-methyl-d-aspartate receptor (NMDAR) antagonist. The mechanism of the antidepressant effect of ketamine is still not fully understood. Studies have indicated that it involves the inhibition of presynaptic and postsynaptic NMDARs in GABAergic interneurons. This effect causes a disinhibition of glutamate transmission and the subsequent glutamate release activates ionotropic α-amino-3-hydroxy-5-methyl-4-isoxazolepropionic acid (AMPA) receptors, and the brain-derived neurotrophic factor-tyrosine kinase receptor B (BDNF-TrkB) signaling pathway, thus, leading to the release of BDNF. Ketamine elicits the activation of neuronal connection through the (long-term potentiation) LTP-like enhancement of glutamatergic synapses, increasing synaptic plasticity (9, 10).

Anhedonia involves various neural circuits, mostly in the reward system of the brain. Studies on anhedonia both in rodents and humans indicate that dopaminergic and glutamatergic signaling is responsible for this symptom (11). A meta-analysis of 11 studies has shown that acute ketamine administration leads to DA release in the brain and increases DA levels in the striatum, nucleus accumbens, and the prefrontal cortex, and correlates with significant increases in DA neuron activity in rodent models of depression, although primate and human studies were inconsistent (12). Ketamine is also a partial agonist of the dopamine D2 receptor (13). A human study using PET found a decreased ability of dopamine D2 receptors to bind (11C)raclopride after s-ketamine administration, indicating increases in the striatal dopamine levels (14). Preclinical evidence suggests that glutamate may have a role in anhedonia (15). Taken together, these findings suggest that the glutamatergic system and its downstream modulation of dopaminergic activity may be one potential route of the antianhedonic efficacy of ketamine in both unipolar and bipolar treatment resistant depression (TRD). Limited evidence on the effect of IV ketamine on anhedonia in humans supports this hypothesis (16–18).

Here, we present the changes in the severity of anhedonia with SHAPS in the course of eight intravenous ketamine infusions in patients with treatment-resistant depression. We hypothesized that subsequent ketamine infusions would improve depressive symptoms and anhedonia and cause a decrease in the SHAPS and IDS 30-SR scores. We also expected that this effect would be smaller in patients taking benzodiazepines.

## Materials and Methods

### Patients

The study population includes subjects enrolled in a naturalistic observational registry protocol for intravenous ketamine treatment in TRD: A Naturalistic Study of Ketamine for Treatment Resistant Mood Disorders (GDKet) (NCT04226963). Detailed methodology is discussed elsewhere (19). The present study comprises a population of 41 patients (26 females) with a mean age of 48.5 years (SD 14.3), with depressive episodes without psychotic features in the course of a major depressive disorder or bipolar disorder. The diagnosis was established by a clinician psychiatrist according to the Diagnostic and Statistical Manual of Mental Disorders (DSM-5) criteria and certified using a Mini International Neuropsychiatric Interview (MINI). All participants exhibited treatment resistance for the current episode. For unipolar depression, for all MDD patients, it was defined as an inadequate response to two or more antidepressants used in adequate doses and time duration according to the Antidepressant Treatment Response Questionnaire ATRQ (20). TRD-BP was defined as a clinically unsatisfactory response to at least two approved dissimilar medications administered in adequate doses and for a sufficient amount of time (21).

Only medically stable adults (<65 years) were enrolled in the study. Medical stability was based on physical examination, medical history, life factors, laboratory tests, and electrocardiography (ECG). If necessary, patients continued current medications during the ketamine treatment. Exclusion criteria included pregnancy, breastfeeding, an active history of uncontrolled diseases, or previous adverse effects while on ketamine. The study protocol was approved by the institutional review board NKBBN/172-674/2019. All participants signed a consent form after confirming their understanding of the study procedures. The study was conducted in accordance with the latest version of the Declaration of Helsinki.

### Study Design

The study followed an observational design; all patients continued baseline psychotropic treatment, as well as necessary treatment of chronic somatic diseases during ketamine infusions. The therapeutic intervention included eight intravenous ketamine infusions administered over 4 weeks as an add-on treatment. Ketamine was administered at a dose of 0.5 mg/kg based on the actual body weight of the patient and given as an intravenous infusion over 40 min. The preparation used for preparing infusions was Ketalar 50 (ketamine hydrochloride) 50 mg/ml; one vial contained 10 ml. The managing psychiatrist monitored safety before, during, and after the infusion every 15 min to 1.5 h after the infusion, including periodic assessment of vital signs (heart rate, body temperature, respiration rate, blood pressure, and oxygen saturation). Safety monitoring included also the Brief Psychiatric Rating Scale (BPRS) and Clinician-Administered Dissociative States Scale (CADSS) at baseline and 1 h after the infusion.

### Psychometric Measures

Anhedonia was measured with the Snaith–Hamilton Pleasure Scale (SHAPS), and a 14-item self-reported measure of anhedonia was used. The items assess anhedonia on a 1–4 scale ranging from “strongly agree” to “strongly disagree.” The SHAPS score can range from 0 to 14, where a score higher than 2 indicates the presence of anhedonia (22). Depressive symptoms were monitored with the 30-item Inventory for Depressive Symptomatology—Self Report (IDS-SR 30) (23). We determined the effect of the change in the level of anhedonia based on the severity of the measured depressive symptoms. The primary outcome was the change in the SHAPS score. The secondary outcomes were the effect of this change on the severity of depressive symptoms measured by IDS-SR 30 and the change in the score of item 18 in the IDS-SR 30 reflecting the intensity of suicidal thoughts. Item 18 describes “Thoughts of death or suicide,” and scores from 0—I do not think of suicide or death, through 1—I feel that life is empty or wonder if it is worth living, 2—I think of suicide or death several times a week for several minutes, up to 3—I think of suicide or death several times a day in some detail, or I have made specific plans for suicide or have actually tried to take my life. The results reported here were assessed before treatment, at the third, fifth, and seventh infusions, and 1 week after treatment.

### Statistical Analysis

Data were analyzed by using the IBM SPSS Statistics package ver. 26. Demographic and clinical characteristics were presented as mean and standard deviation or frequencies. In some cases, other statistics were provided. The normality of the continuous variables was examined by the Shapiro–Wilk test. In addition, the following methods were used: (a) a one-way ANOVA with repeated measures for the results of the SHAPS and IDS-30 complemented with Tuckey's *post-hoc* tests, (b) general linear models with repeated measures for the results of the same questionnaires, adjusted for potential confounders (sex, age, BMI, benzodiazepines), (c) the Friedman's test (following Dunn's *post-hoc* tests) for analysis of IDS-30 item no. 18 scores, and (d) a moderated mediation model for the relationship between ketamine infusions and depression severity (IDS-30) with anhedonia (SHAPS) as the potential mediator and the therapy of benzodiazepines as the moderator of association between the ketamine and SHAPS scores. In order to conduct the above analysis, we used PROCESS macro (v3.5) for SPSS, following the bootstrapping procedure with 5,000 resamples. All calculations were made for a sample of *N* = 41. The significance level was set at α = 0.05. With the assumed moderate effect for the selected methods, the analyzed sample (*N* = 41) allowed to obtain the power of the test at a level > 0.8.

## Results

### Demographic and Clinical Analysis

Demographic and clinical analysis of the study sample is presented in Table 1.

**Table 1:** Demographic and clinical characteristics of the total sample.

**Variables**	**Total sample (*N* = 41)**
	**Mean (SD)**
Age	48.5 (14.3)
BMI	27.5 (5.6)
Episode duration (weeks)	26.9 (29.0)
Number of depressive episodes	6.1 (8.1)
**Baseline results (before infusion)**	
SHAPS	9.6 (3.5)
IDS-30 total	46.7 (13.1)
IDS item no. 18	1.2 (1.2)
**Diagnosis**	*N* (%)
MDD	28 (68.3%)
BD	13 (31.7%)
**Sex**	
Females	26 (63.4%)
Males	15 (36.6%)
**Education**	
Primary	2 (4.9%)
Secondary	4 (9.8%)
Vocational	16 (39.0%)
Higher	19 (46.3%)
**Employment status**	
Unemployed	8 (19.5%)
Pension	16 (39.0%)
Retirement	7 (17.1%)
Employed	9 (22.0%)
Study	1 (2.4%)
**Marital status**	
Single	11 (26.8%)
Informal relationship	2 (4.9%)
Married	20 (48.8%)
Divorced	5 (12.2%)
Widowed	3 (7.3%)
**Concomitant meds**	
TCA	MDD: 4 (14.3%), BD: 0 (0%)
SSRI	MDD: 16 (57.1%), BD: 4 (30.8%)
SNRI	MDD: 5 (17.9%), BD: 5 (38.5%)
Other	MDD: 11 (39.3%), BD: 3 (23.1%)
Antipsychotics	MDD: 8 (28.6%), BD: 8 (61.5%)
Mood stabilizers	MDD: 10 (35.7%), BD: 10 (76.9%)
Lithium	MDD: 0 (0%), BD: 3 (23.1%)
BDZ	MDD: 12 (42.9%), BD: 8 (61.5%)

*SHAPS, Snaith–Hamilton Pleasure Scale; IDS-SR 30, 30-Item Inventory for Depressive Symptomatology—Self Report; SD, standard deviation; BMI, body mass index; MDD, major depressive disorder; BD, Bipolar disorder; SSRI, selective serotonin reuptake inhibitors; SNRI, serotonin and noradrenaline reuptake inhibitors; AD, antidepressants; TCA, tricyclic antidepressants; BDZ, benzodiazepines*.

We did not observe any serious adverse effects. Patients experienced a mild and transient increase in arterial blood pressure and self-limiting mild-to-moderate dissociative symptoms.

### Snaith–Hamilton Pleasure Scale

**Figure 1:**
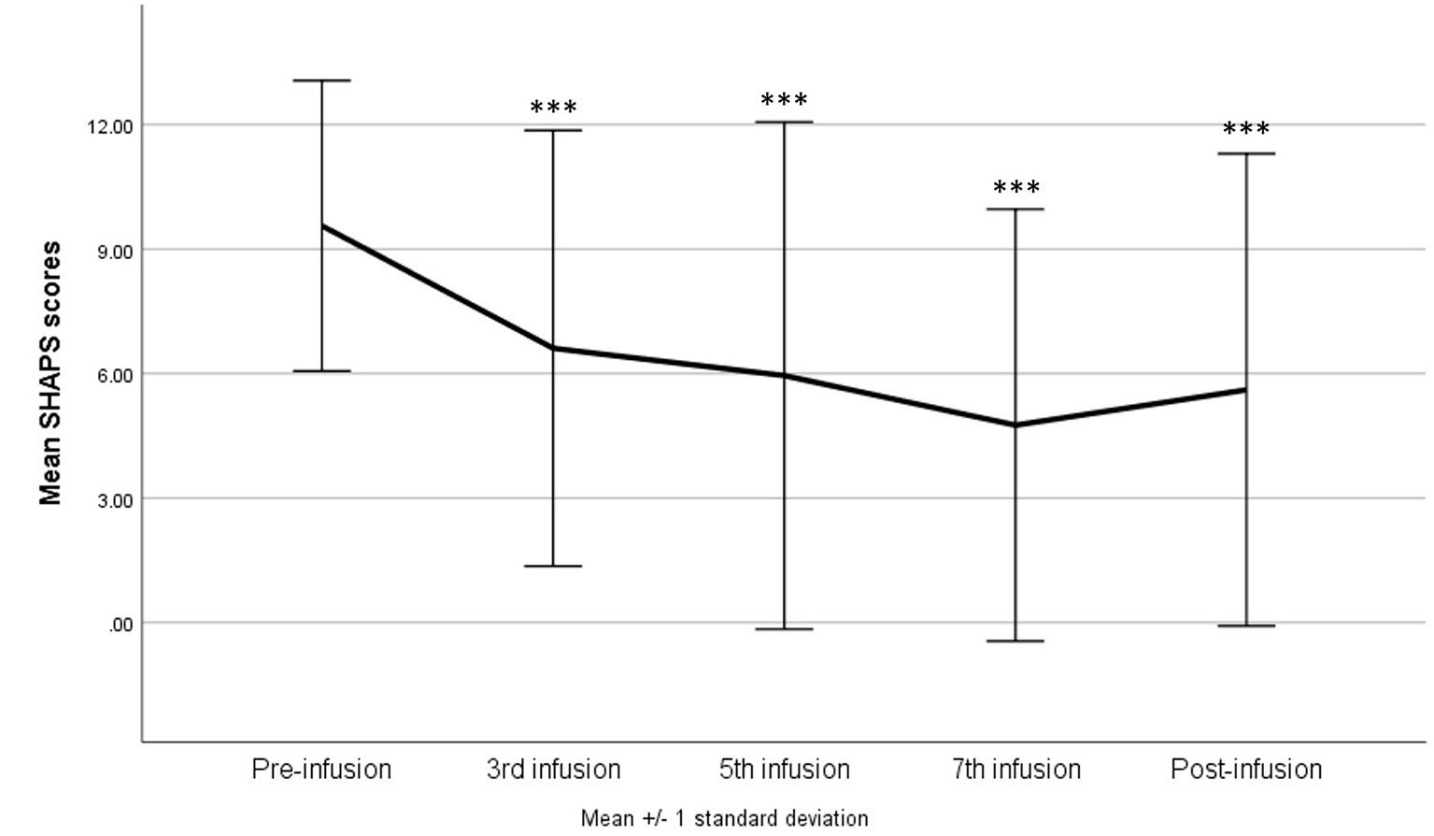
Snaith–Hamilton Pleasure Scale (SHAPS) score change during ketamine treatment. ***p < 0.001 in comparison with baseline scores (pre-infusion).

At baseline, 97.5% of the patients (*n* = 39) met the criteria for clinically significant anhedonia (i.e., SHAPS ≥ 2). The simple ANOVA with repeated measures indicates a significant reduction in the SHAPS total score across infusions—*F*_(4,160)_ = 13.36, *p* < 0.001, η^2^*p* = 0.25; with G-G and H-F correction *p* < 0.001. Pairwise comparisons (Tuckey's *post-hoc* tests) show that there was a significant decrease in the SHAPS total score from the baseline to each infusion and the post-infusion visit (in all comparisons *p* < 0.001) (Figure 1). No significant differences emerged between each infusion and between infusions and the post-infusion visit.

**Figure 2:**
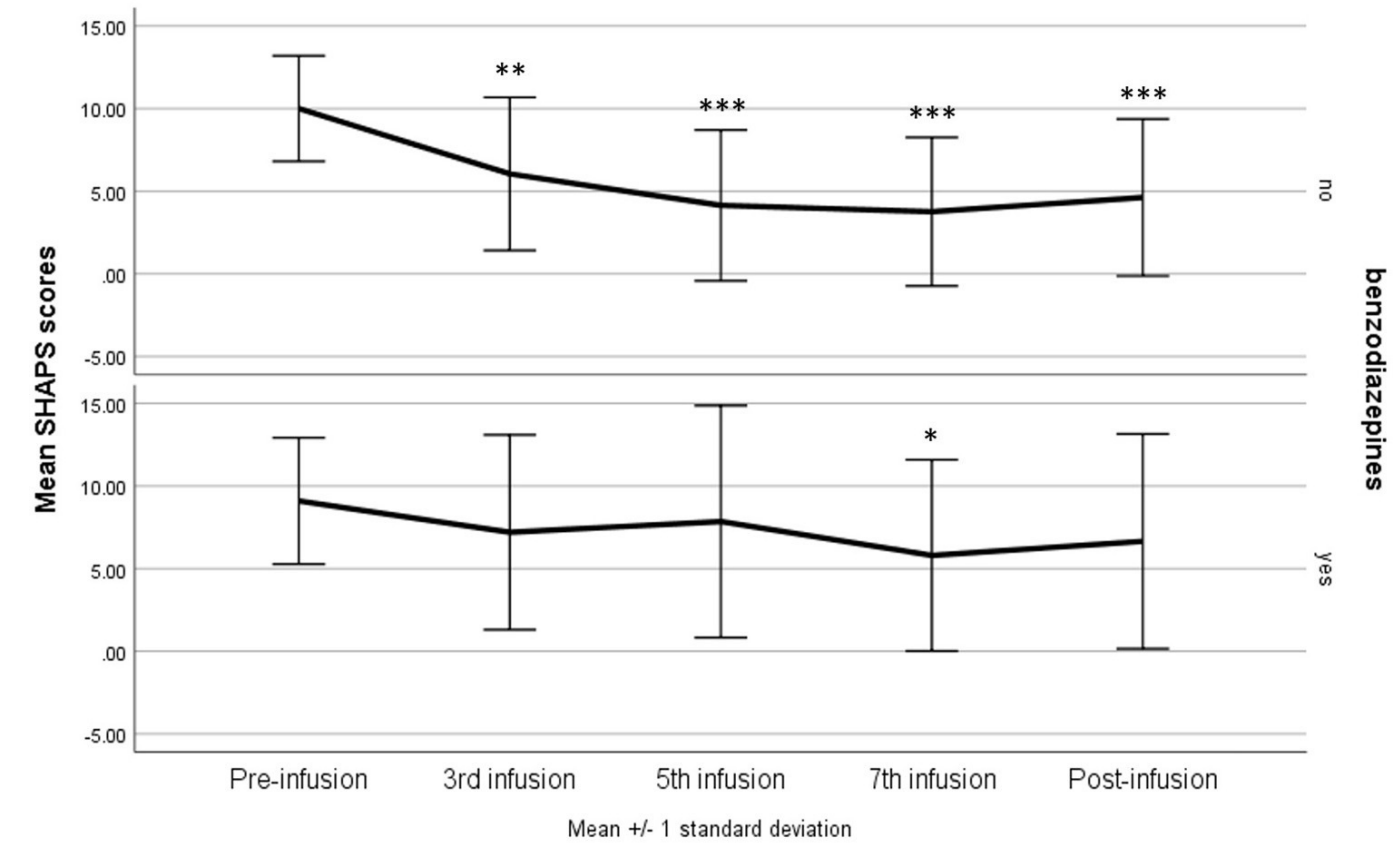
Snaith–Hamilton Pleasure Scale (SHAPS) score change during ketamine treatment in patients using benzodiazepines compared with the rest of the study group. *p < 0.05, **p < 0.01, ***p < 0.001 in comparison with baseline scores (pre-infusion).

After adjusting for potential confounders (sex, age, BMI, and treatment with benzodiazepines), there was a significant interaction between the ketamine infusion and use of benzodiazepines—*F*_(4, 144)_ = 2.60, *p* = 0.039, η^2^*p* = 0.07 with H-F correction *p* = 0.041. A significant reduction in the SHAPS after each infusion (*p* = 0.002 in comparison with the third infusion and *p* < 0.001 in comparison with the rest infusions) and during the post-infusion visit (*p* < 0.001) was observed only among patients not using benzodiazepines. In the second group, a reduction (in comparison with baseline results) was noticed only after the seventh infusion (*p* = 0.036); however, eventually, the total score did not differ between the baseline and the post-infusion visit (Figure 2).

### 30-Item Inventory for Depressive Symptomatology—Self Report

**Figure 3:**
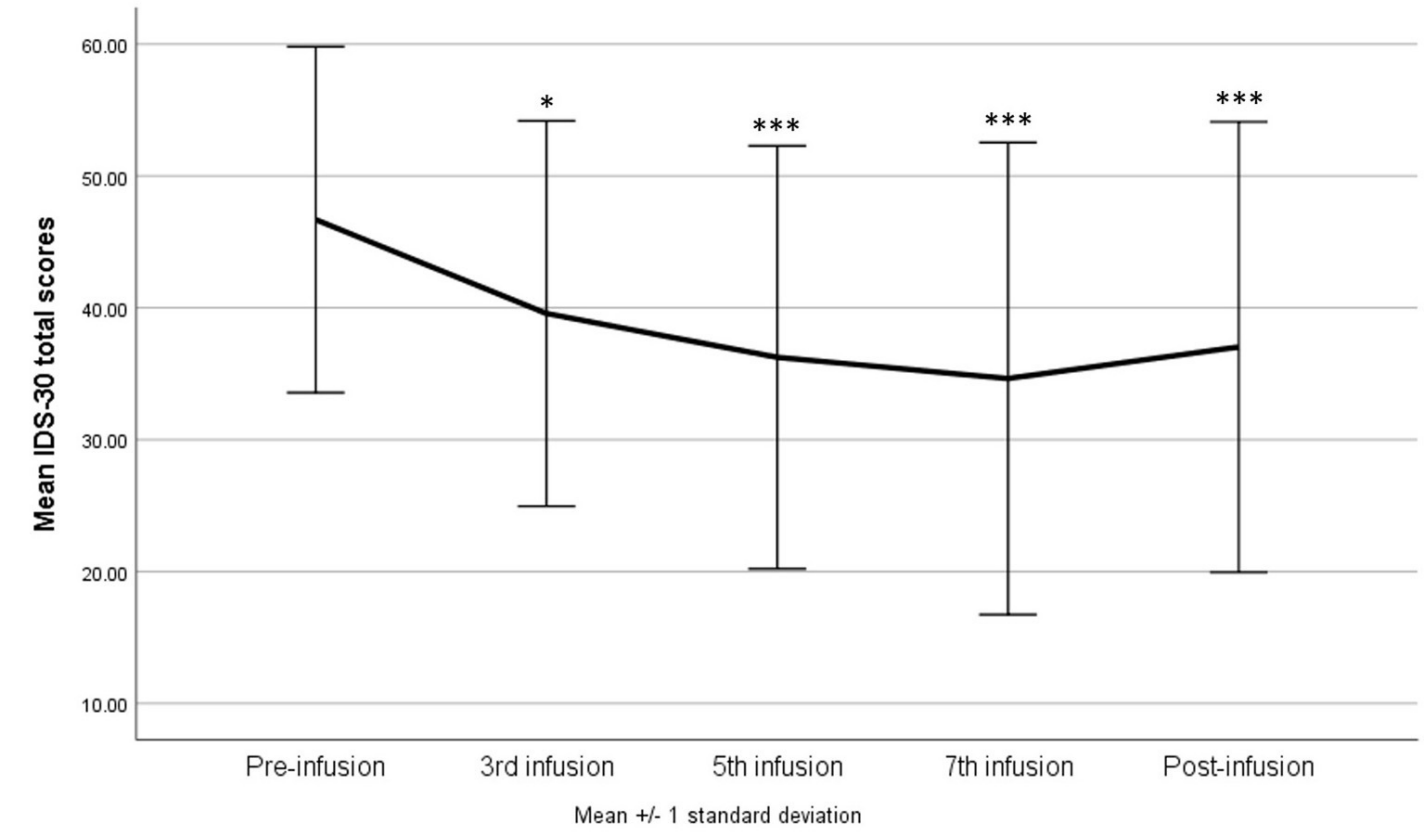
The 30-item inventory for Depressive Symptomatology—Self Report (IDS-SR 30) score during ketamine treatment. *p < 0.05, ***p < 0.001 in comparison with baseline scores (pre-infusion).

The same models as above were tested for the IDS-30 total scores as dependent variable. In the one-way repeated measures of ANOVA, the result was statistically significant—*F*_(4, 160)_ = 8.86, *p* < 0.001, η^2^*p* = 0.18; with G-G and H-F correction *p* < 0.001, suggesting a gradual decrease in the score as a result of the administering of ketamine. The difference between the baseline and third infusion was significant at *p* = 0.013, and between the baseline results and subsequent infusions and the post-infusion visit *p* < 0.001 (Figure 3). The model adjusted for confounding variables indicated a sustained statistically significant effect of the infusions—*F*_(4, 144)_ = 3.28, *p* = 0.013, η^2^*p* = 0.08; with G-G and H-F correction *p* < 0.05, but no significant interaction effects.

### 30-Item Inventory for Depressive Symptomatology—Self Report 30 Item No. 18

Due to the nature of the variable, the analysis of the changes in scores was carried out using the non-parametric Friedman test (and *post-hoc* Dunn's test), while the model taking into account confounding variables was omitted. The results showed a significant decrease in the intensity of suicidal ideation as a result of ketamine infusions [$\chi_{\left(4\right)}^{2}$ = 15.53, *p* = 0.004, *W* = 0.09], but this change was observed only after the fifth (*p* = 0.047) and the seventh (*p* = 0.028) infusions in comparison with the pre-infusion measurement. At the same time, the results obtained during the post-infusion visit did not differ from the baseline. Instead of a graph, a table with descriptive statistics is presented for better readability.

### Mediation Model

**Figure 4:**
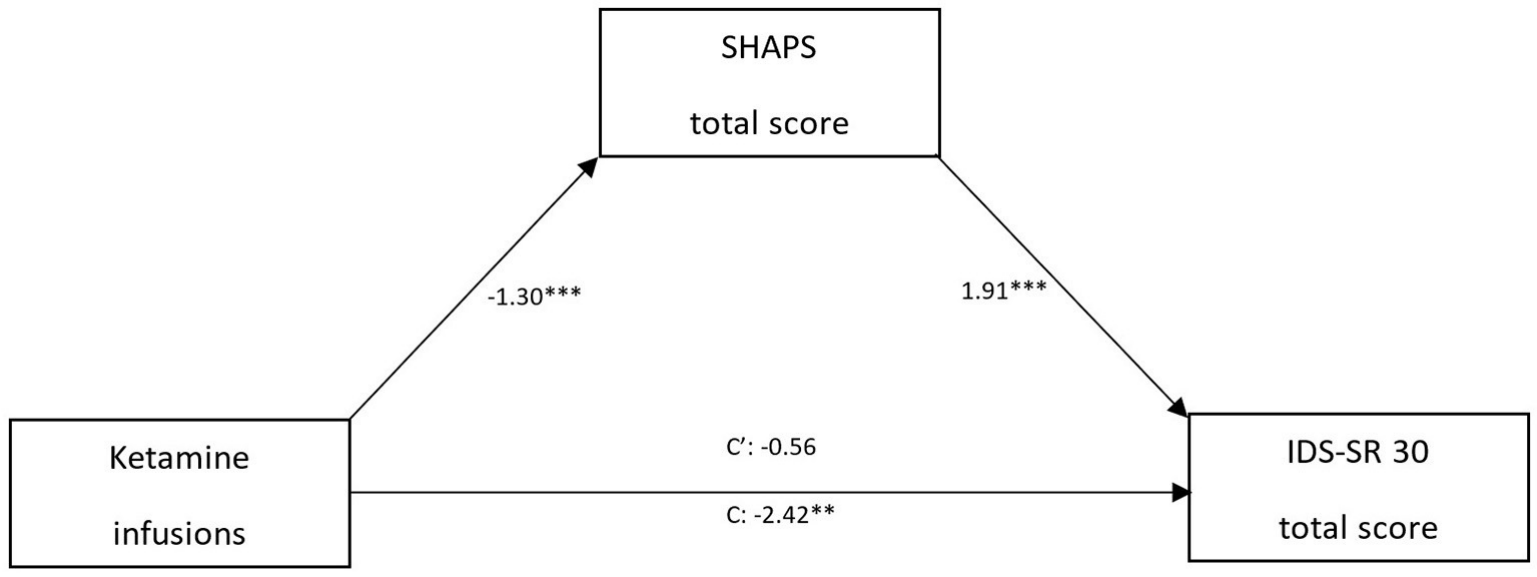
The mediating effect of anhedonia in the relationship between ketamine treatment and the 30-item Inventory for Depressive Symptomatology—Self Report (IDS-SR 30) score. Snaith–Hamilton Pleasure Scale (SHAPS). ^#^The model presents unstandardized effects.

Mediation analysis with moderation (model 7. in macro PROCESS v3.5.) was conducted in order to determine if there was an indirect effect of anhedonia on the relationship between the number of infusions and the severity of depression. Taking benzodiazepines was included as a moderator of the relationship between infusions and the SHAPS total scores. Due to the lack of significance of the interaction between infusions and benzodiazepines in the overall model, the mediation model without a moderator was finally analyzed (model 4. in macro PROCESS v3.5) (Figure 4). The obtained results indicate a significant unstandardized indirect effect of anhedonia in the relationship between infusions and the severity of depression: −1.90 (95% CI bootstrap method: −2.83 to −0.91). The whole model was significant—*F*_(2, 202)_ = 74.42, *p* < 0.001 and explained 42% of the variance of the dependent variable.

## Discussion

A primary finding in this study is a statistically significant decrease in the level of anhedonia during ketamine treatment. It was also found that significant reduction in SHAPS after each infusion and 1 week post-treatment was observed only among patients not using benzodiazepines. The IDS-SR 30 score reduced significantly during treatment. We observed a significant decrease in the intensity of suicidal ideation reflected in the score of item 18 after the fifth and the seventh infusions. The indirect effect of anhedonia on the severity of the depression was investigated in a mediation model, which confirmed that reduction in depressive symptoms was mediated by the antianhedonic effect, meaning that the general level of depression is reduced due to a decrease in the symptoms of anhedonia. The change in the level of anhedonia explained 42% of the variance of the IDS-SR 30 score improvement.

The results are in line with the existing evidence, although data on the effect of ketamine on anhedonia are scarce. One study has shown a rapid antianhedonic effect after a single infusion of ketamine in 36 patients with treatment-resistant bipolar depression (16). A subsequent open label study by the same group included 52 participants with TRD who received one infusion of 0.5 mg/kg ketamine after a 2-week washout period, which also confirmed a rapid antianhedonic effect of ketamine (17). In a study investigating the clinical correlates of suicidal thoughts, the authors found that after a single infusion of ketamine, TRD patients with MDD and BD significantly reduced their SHAPS scores. The authors also observed that improvements on the SHAPS accounted for an additional 13% of the variance in suicidal thought reduction, beyond the influence of depressive symptoms (24). A recent retrospective *post-hoc* analysis of over 200 participants with treatment-resistant depression in the course of MDD or BD revealed a significant reduction in the SHAPS score during and after four ketamine infusions. The authors also found that improvements in depressive symptoms, suicidal ideation, and anxiety symptoms were mediated by a reduction in anhedonic symptoms since anhedonia accounted for 26% of the variance in depressive score improvements measured with QIDS 16 (18).

Our secondary finding was that there appeared to be no significant effect of ketamine on anhedonia in the subgroup of patients using benzodiazepines compared with the rest of patients. There is some evidence on the attenuation of the antidepressant effect of ketamine by concomitant BDZ. One case report describes a patient with bipolar depression treated with 10 infusions of ketamine as an add on to lithium, fluoxetine, quetiapine, and 3.5 mg of lorazepam. During lorazepam administration, the response to ketamine was limited to 24 h, but when lorazepam was withdrawn, the response extended to 10–14 days. No other changes in medications were made (25). A *post-hoc* analysis of data of 10 TRD patients treated with ketamine has shown that the responder group had a significantly smaller dose of BDZ used than a non-responder group (26). Another *post-hoc* analysis of the effect of six ketamine infusions on 13 TRD patients has shown that BDZ users had a longer time to response and remission as well as a shorter time to relapse compared with the rest of the group. A recent *post-hoc* analysis of data from 47 MDD patients treated with a single infusion of ketamine has shown significantly worse outcomes in the subgroup receiving BDZ, and this effect depended on the dose of the BDZ. Moreover, the BDZ attenuating effect was present only on days 3 and 7, not at 24 h post infusion, which suggests that it may be related to the neurotrophic effect of ketamine treatment (27). Our results stand in line with the aforementioned reports, although in the cited papers, the investigators focused on the severity of depression, and our objective was to study this effect on anhedonia. The process of interference of BDZ on the ketamine effect can be hypothetically explained as follows: ketamine blocks NMDA receptors causing disinhibition of GABAergic interneurons and a subsequent increase in glutamatergic activity with a following BDNF increase. Benzodiazepines act as agonists against GABA-A receptors and, therefore, counteract the effect on the GABAergic neurons stimulating them.

Ketamine and benzodiazepines have a common target, which is the reward system. Dysregulation of the reward system function has been described in depression, anhedonia, but also in addictions (28). Despite being very useful in short-term treatment for anxiety, there is evidence that benzodiazepines might worsen depressive symptoms (29). In a study including over 900 TRD patients, the authors found that regular benzodiazepine use was a strong correlate of treatment resistance. The suggested reason is the possible coexistence of anxiety disorder, suppression of feelings as an effect of BDZ, which can deepen depression and undermine the effectiveness of psychotherapy as well as the negative influence of BDZ on cognitive functions (30). The same group conducted a study on 1,034 (128 BP I, 906 BP II) patients with treatment for bipolar depression, dividing them into three groups: low, medium, and high resistance, based on the number of medications they used. The authors found that regular BDZ use was significantly more common in the last group, defined as the TRBD group using five or more psychotropic medications (31).

Similar to benzodiazepines, ketamine has abuse potential (32); therefore, it is particularly important to follow-up patients on ketamine treatment, especially on long-term use as this regimen seems to be the most effective (33).

Several limitations of the study should be taken into consideration. The study was conducted without a placebo control, randomization, and blinding. Thus, the results apply to the naturalistic observational design. Apart from these obvious shortcomings, the registry design has also benefits and can help to study the effect of medication in a real-life setting (34). There were no measurements of ketamine and its metabolites as well as the concentrations of benzodiazepines in the blood of patients. Another limitation is that unipolar and bipolar patients were included in the same group due to the relatively small number of participants. This was also the reason why we did not analyze different benzodiazepines separately. Observational studies provide an overall low quality of evidence, and the true effect might be markedly different from the estimated effect due to the risk of bias, imprecision, inconsistency, and indirectness, and the present level C of evidence that is insufficient for scientific recommendation. However, our report aims at contributing to the literature with regard to the matters of safety and tolerability, which may be useful for future research with a more rigorous design.

In summary, our study results suggest that there is an antianhedonic effect of intravenous ketamine in patients with treatment-resistant depression in the course of unipolar and bipolar disorder in an open label naturalistic study.

This effect was attenuated by the use of benzodiazepines. The use of ketamine needs to be monitored in order to prevent the development of substance use disorder. Our findings need replication in a large placebo controlled RCT.

## Data Availability Statement

The raw data supporting the conclusions of this article will be made available by the authors, without undue reservation.

## Ethics Statement

The studies involving human participants were reviewed and approved by Institutional Ethics Committee of Medical University of Gdańsk (NKBBN/172-674/2019). The patients/participants provided their written informed consent to participate in this study after confirming their understanding of the study procedures. The study was conducted in accordance with the latest version of the Declaration of Helsinki.

## Author Contributions

WC and MW: conceptualization. WC: methodology and funding acquisition. MW, AWi, and AWł: software. MW and AWł: formal analysis. MG-W and AWł: investigation. MW: resources. AWi and AWł: data curation. AWi: writing—original draft preparation. AWi and WC: writing—review and editing. MG-W and MW: project administration. All authors have read and agreed to the published version of the manuscript.

## Funding

This work was supported by the Medical University of Gdańsk, Poland (Grant No. ST-02-0039/07/221) and represents data from the clinical registry A Naturalistic Study of Ketamine for Treatment Resistant Mood Disorders (GDKet) (NCT04226963).

## Conflict of Interest

AWi has received research support from Angelini, Biogen, Eli Lilly and Company, Janssen- Cilag, Lundbeck, Polpharma, Sanofi and Valeant. MW has received research support from Acadia, Apodemus, Alkermes, Auspex Pharmaceuticals, Cephalon, Ferrier, Forest Laboratories, Gedeon Richter, GWPharma-ceuticals, Janssen, Lundbeck, Orion, Otsuka, Servier; Speaker s Bureau: Lundbeck, Servier. MG-W has received research support from: Janssen, Servier, Alkermes, KCR, Lilly, Biogen, Celon. AWł has received research support from Actavis, Eli Lilly, Minerva Neurosci-ences, Sunovion Pharmaceuticals, KCR, Janssen, Otsuka, Apodemus, Cortexyme, Acadia. WC has received research support from Actavis, Alkermes, Allergan, Angelini, Auspex, Biogen, Bristol-Myers Squibb, Cephalon, Eli Lilly, Ferrier, Forest Laboratories, Gedeon Richter, GW Pharmaceuticals, Janssen, KCR, Lundbeck, Orion, Otsuka, Sanofi, and Ser-vier; he has served on speakers bureaus for Adamed, Angelini, AstraZeneca, Bristol-Myers Squibb, Celon, GlaxoSmithKline, Janssen, Krka, Lekam, Lundbeck, Novartis, Orion, Pfizer, Polfa Tarchomin, Sanofi, Servier, and Zentiva; and he has served as a consultant for GW Pharmaceu-ticals, Janssen, KCR, Quintiles, and Roche.

## Publisher's Note

All claims expressed in this article are solely those of the authors and do not necessarily represent those of their affiliated organizations, or those of the publisher, the editors and the reviewers. Any product that may be evaluated in this article, or claim that may be made by its manufacturer, is not guaranteed or endorsed by the publisher.
